# Red blood cell ATP/ADP & nitric oxide: The best vasodilators in diabetic patients

**DOI:** 10.1186/2251-6581-11-9

**Published:** 2012-08-24

**Authors:** Nuredin Bakhtiari, Saman Hosseinkhani, Bagher Larijani, Mohammad Reza Mohajeri-Tehrani, Abbas Fallah

**Affiliations:** 1 Department of Biochemistry, Faculty of Biological Sciences, Tarbiat Modares University, Tehran, Iran; 2 Endocrinology and metabolism research center, Tehran university of medical sciences, Tehran, Iran; 3 Department of Exercise-physiology, faculty of Humanity sciences, Tarbiat Modares University, Tehran, Iran

**Keywords:** Diabetes, ATP/ADP, Nitric oxide, Red blood cell

## Abstract

**Background:**

Diabetes mellitus is a group of metabolic diseases characterized by high blood sugar (glucose) levels that result from defects in insulin secretion, or action, or both. Inspired by previous report the release of ATP from RBCs, which may participate in vessel dilation by stimulating NO production in the endothelium through purinergic receptor signaling and so, the aim of this study is to clearly determined relationship between RBC ATP/ADP ratio with nitric oxide.

**Methods:**

The ATP/ADP ratio of erythrocytes among four groups of normal individuals (young & middle age), athletes’ subjects and diabetic patients were compared and the relationship between ATP/ADP ratio and NO level of plasma was determined with AVOVA test and bioluminescence method.

**Results:**

ATP/ADP level in four groups normal (young & middle age), athletes, diabetes] are measured and analyzed with ANOVA test that show a significant difference between groups (P-value < 0.001). A significant positive correlation was found between RBC ATP/ADP content (r = 0.705; P < 0.001). Plasma NO content is also analyzed with ANOVA test which shows a significant difference between groups.

**Conclusion:**

In this study, a positive relationship between RBC ATP/ADP ratio and NO was found. Based on the obtained result, higher RBC ATP/ADP content may control the ratio of plasma NO in different individuals, also this results show that ATP can activate endothelial cells in NO production and is a main factor in releasing of NO from endothelial cells.

## Introduction

Diabetes mellitus is a group of metabolic diseases which interfere with energy homeostasis [1]. Diabetes is one of the most costly chronic diseases with an estimated worldwide prevalence of 170 million in 2002, which is expected to double by 2030 according to the World Health Organization [2]. The most common macrovascular complication of diabetes is atherosclerosis, which increases the risk for myocardial infarction, stroke, and peripheral artery disease, the latter being the leading cause of limb amputation in civilized countries. Microvascular complications consist of retinopathy and nephropathy, the leading causes of blindness and renal failure [3]. There is now substantial evidence indicating that endothelial dysfunction, characterized by diminished endothelium dependent relaxation, is abnormal in experimental models of diabetes [4-8]. Several other studies have also demonstrated impaired endothelium dependent vasodilation in human diabetic patients [9,10]. There is substantial evidence suggesting that high concentrations of glucose could reduce NO availability due to an increase in superoxide anion production [11]. Further, elevated glucose levels may lead to a decrease in cellular concentrations of nicotinamide adenine dinucleotide phosphate (reduced form) (NADPH) through activation of the polyol pathway [12]. As NADPH is an essential cofactor of NO synthase (NOS) for NO synthesis, its depletion could lead to a reduction in endothelial NO production. NO mediates the endothelium-dependent vasodilation in response to stimuli such as shear stress, insulin, and acetylcholine [13]. Recent evidence suggests that NO interacts with other flow-induced vasodilation mediators, including prostacyclin and adenosine, and this interaction results in net hemodynamic changes. NO has also been shown to mediate exercise-induced vasodilation [14]. As a potent endogenous vasodilator, NO has numerous beneficial effects that preserve normal vascular function. For example, NO activates guanylate cyclase in vascular smooth muscle, and increases the production of cyclic guanosine monophosphate (cGMP). cGMP, as a second messenger, mediates many of the biological effects of NO, e.g. causing relaxation of smooth muscle and inhibiting platelet aggregation [15]. In addition, NO inhibits platelet activation and adhesion to the surface of endothelium. NO reduces vascular oxidative stress and inhibits superoxide generation [16,17]. In addition, NO stimulates angiogenesis, which plays an important role in wound healing, vascular remodeling, and conditions like myocardial infarction and diabetic retinopathy [18]. NO is also a mediator of the immune response, a neurotransmitter, a cytotoxic free radical and a widespread signaling molecule [16,18]. NO stimulates insulin release from pancreatic ß cells in the presence of glucose [19]. These properties suggest that the level of NO production by the endothelium may play a pivotal role in the regulation of vascular disease. Therefore, impaired endothelial NO production may constitute a critical manifestation of proatherogenic events in the vascular wall, including increased vascular tone, platelet aggregation, endothelial barrier dysfunction, vascular inflammation, and smooth muscle cell proliferation. Nisoli et al [20] recently reported that the NO-cGMP-dependent pathway controls mitochondrial biogenesis and body energy balance. For example, NOS null mutant mice had a reduced metabolic rate and accelerated weight gain [20], insulin resistance [21], hypertension, and hyperlipidemia [22]. Accordingly, it was reported that the red blood cell (RBC) is a determinant of endogenous nitric oxide (NO) synthesis in the pulmonary circulation; i.e., in the isolated perfused rabbit lung, RBCs obtained from either rabbits or healthy humans were required component of the perfusate in order to demonstrate flow-induced endogenous NO synthesis [23]. RBCs contain concentrations of ATP adequate to activate the endothelial P2y purinergic receptors, resulting, thereby, in synthesis of NO [24,25]. Importantly, the component of blood, responsible for the stimulation of endogenous NO synthesis, was determined to be the RBC, via the release of adenosine triphosphate (ATP) [26]. Indeed, the application of ATP to endothelial cells resulted in an increase in NO synthesis [24,27]. ATP is of particular interest because it is present in millimolar amounts in RBCs [28]. In the present study, we investigated the RBC ATP/ADP ratio and plasma nitric oxide in different groups (normals, diabetes). We characterized a significant coherency between ATP/ADP and nitric oxide .in addition, in this study we determined these factors in athletics’ subject, all of them were jodo national team. Finally, to bold the results, they comparison with each other.

## Materials and methods

In this study we investigated on human subjects*,* all control subjects were healthy volunteer living in the community, and none was acutely ill. None exhibited evidence of cardiac or chronic kidney disease and all were euthyroid with normal liver function tests and normal value for plasma urea, creatinine and electrolytes. Furthermore, healthy subjects were drug free and with a negative family history of diabetes mellitus or hypertension. The subjects were fully informed of any risks and discomforts associated with the experiments before giving their informed written consent to participate. The studies conformed to the code of Ethics of the World Medical Association (Declaration of Helsinki) and were approved by the Ethics Committee of Shariati Hospital, Tehran University of Medical Science of Iran. RBC of 40 uncontrolled diabetes patients was collected (40-65 years) and at the time of the study (HbA1C% 8.79 ± 0.19) we also collected RBC from 60 healthy volunteer (23-55 years), this group was divided into three classes, normals [young individuals ( 20-33 years) and middle age subjects ( 40-55 years)] and athletes (20-33 years). The following parameters were determined in all blood samples which collected at early morning after overnight fast: erythrocyte ATP, ADP content and plasma nitric oxide (NO) level.

### ATP assay method in RBC

ATP was measured by luciferin-luciferase technique [29,30]. In which the amount of light generated by the reaction of ATP with recombinant luciferase is dependent on the ATP concentration. Sensitivity was augmented by addition of the D-luciferin to the luciferase. A, 50 μl sample of RBC, lysed with TCA 10% (tri*coloro*aceticacid) and neutralized with KOH 1 M and diluted with hepes buffer 100 mM pH 7.8 (1:64), injected into a cuvette containing 10 μl luciferin (sigma), 10 μl Mgso4, 10 μl luciferase(1 mg/ml). The peak light efflux from cuvette to which either known ATP standards or samples are added was determined using a luminometer (Sirius tube Luminometer, Berthold Detection System, Germany), a ATP standard curve was obtained on the day of each experiment.

### RBC ADP assay procedure

ADP was measured by the coupled assay of pyruvate kinase with luciferin-luciferase technique [31], in which at first we injected 5 μl pyruvate kinase (1 mg/ml) into a cuvettes containing 50 μl RBC (lysed, neutralized and diluted), 5 μl PEP (phospho enol pyruvate 20 mM, sigma), 5 μl KCl and patience for 7 min since the ADPs existed in the sample converted to ATP, then added 10 μl luciferin (10 mM), 10 μl luciferase (1 mg/ml) and 10 μl Mgso_4_. The peak light efflux from cuvette to which either known ADP standards or samples are added was determined using a luminometer, an ADP standard curve was obtained on the day of each experiment.

### NO assay procedure

NO was measured by the dye assay based on Griess reaction [32]. In this study to inhibition of blurred solution which interferes with assay we used Znso4 as a protein degenerative (solution clarification) [33,34]. In this assay which act based on Griess reaction, added 100 μl a mixture of solution, NEDD (N-1-naphtylethylenediamine) and sulphanomide (1:1) to 100 μl of clarified serum, to doing reaction and dye forming, solution mixture placed into incubator 37°C for 30 min. after this time dye formed from this reaction measured at 540 nm by Elisa reader ( Sunrisa, Tecan, Austrian).

### Statistical method

Statistical significance among experimental periods and groups was determined with analysis of variance, Tamhane test and Scheffe test for multiple comparisons, Bivariate correlation for relation between variables and regression for prediction. A P-value 0.05 or less was considered statistically significant. Results are reported as the means ± SEM.

## Results

### Human subject studied

RBCs are taken from 40 male diabetic patients who were diagnosed according by American Diabetes Association Guideline 2011 [35]. The average age of the patients was 60.5 ± 1.7 years. Baseline characteristic data are summarized in Table 1. In addition, RBC of 60 healthy human volunteers [20 athletes, 40 Normals (20 youngs, 20 middle age)] with equal sex for all was sampled. Average ages for athletic individuals without medication and history of diabetes disease, was 27.7 ± 0.61 and for normal control was 26.7 ± 0.49 and 50 ± 0.60.

**Table 1:** Age and sex characteristics of studied subjects: Erythrocyte ATP/ADP and plasma NO content, Tamhan test for ATP/ADP group comparison, as shown in this table the difference between groups are significant (P-value <0.001)

	Normal(Y)*	Normal(M)**	**Athletes**	**Diabetes**
** N**	20	20	20	20
**Age(year)**	26.7 ± 0.49	50 ± 0.60	27.7 ± 0.61	60 ± 1.7
**Sex**	Male	Male	Male	Male
**BMI**^**a**^	25	28	20	33
**ATP/ADP**^**b**^	3.08 ± 0.09	2.4 ± 0.07	4.96 ± 0.411	1.26 ± 0.08
**NO(ng/dl)**^**c**^	38.27 ± 1.4	31 ± 1.65	50.58 ± 1.33	25.37 ± 0.96
**HbA1C%**	4.86 ± 0.13	5.31 ± 0.11	4.09 ± 0.08	8.79 ± 0.19
**FBS**^**d**^	84.05 ± 2.5	102.5 ± 2.7	76 ± 0.94	172 ± 5.9

a = Body Mass Index [weight/(height)^2]^ = kg/m^2^, b = P value < 0.001 c = P value < 0.001 d = fasting blood sugar * Y = young, ** M = middle age

Scheffe test for comparison between groups shows that NO differences between normal(M) and diabetes individuals are not significant.

### ATP/ADP level between groups

**Figure 1:**
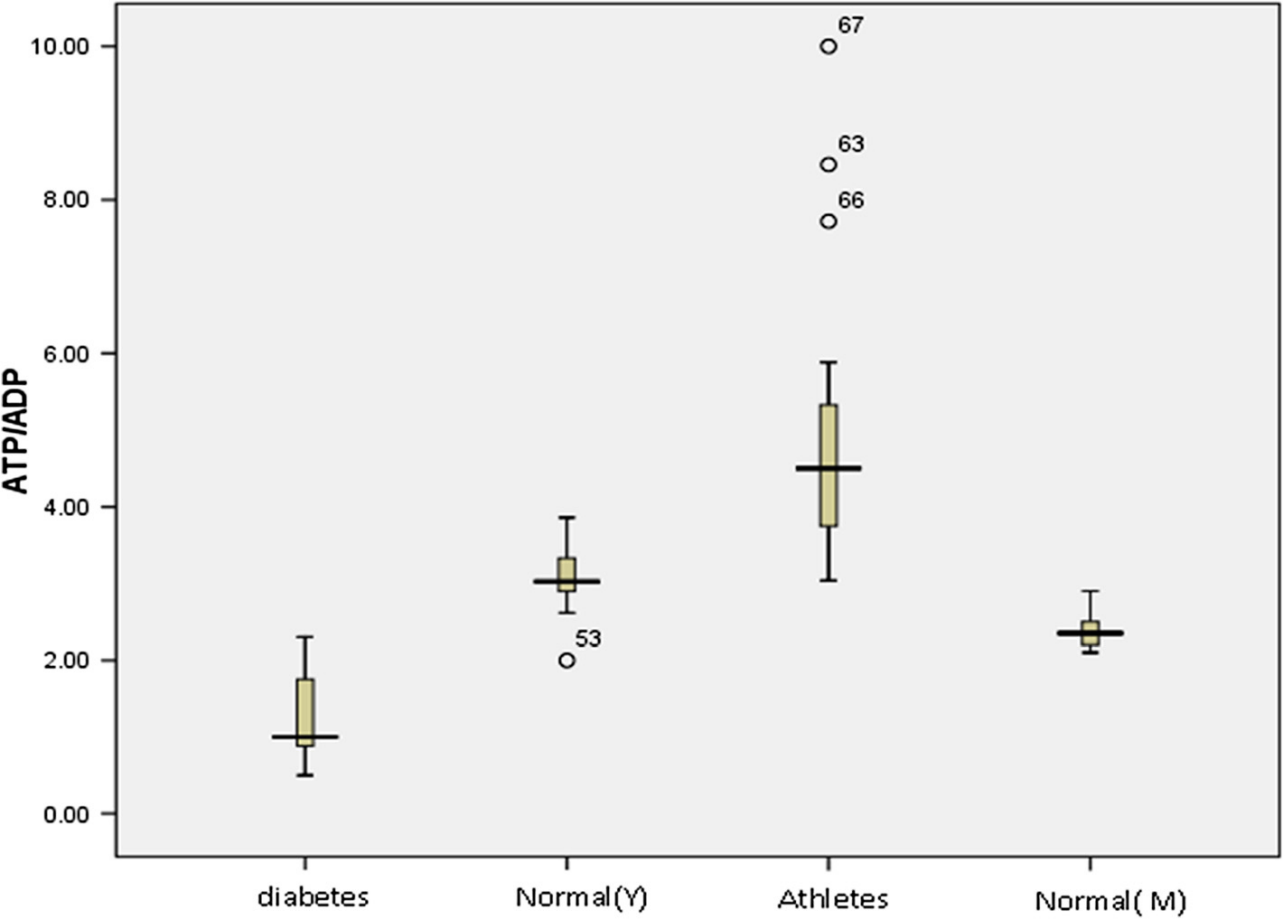
The comparison of ATP/ADP level among 4 groups (diabetes, Normal (Y = young, M = middle age) and athletes.

In this study the control individuals divided to three groups, normal (young) & normal (middle age) and athlete individuals. ATP/ADP level in four groups (Normal (Y) & Normal (M), athletes, Diabetes) was measured and analyzed with ANOVA test, the result showed a significant difference between groups (P value < 0.001), the result are summarized in Table 1 and Figure 1.

### NO level between groups

**Figure 2:**
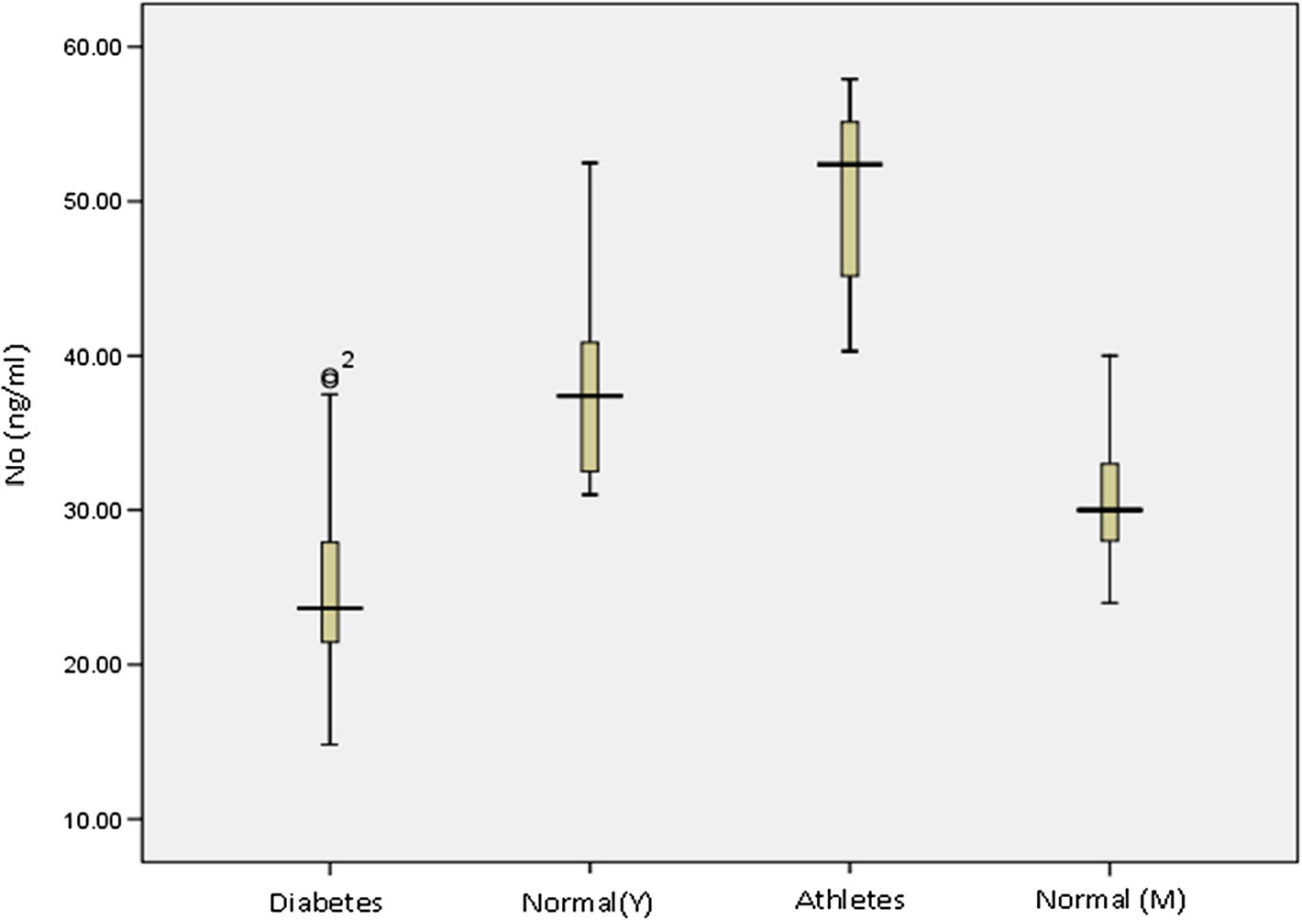
The comparison of NO level among 4 groups (diabetes, Normal (Y = young, M = middle age) and athletes.

In this study we measured NO by dye assay and result was analyzed with ANOVA test. As the difference variance between groups was not significant, data were analyzed with fisher and Scheffe test. The results are summarized in Table 1 and Figure 2.

### Correlation between RBC ATP/ADP content and NO

A significant positive correlation was found between RBC ATP/ADP content (r = 0.705; P < 0.001). In this study, a positive relationship between ATP/ADP and NO is found, accordingly; 0.705 unit increase in NO is accompanied by 1 unit increase of ATP/ADP ratio. Therefore, based on experimental measurements reported here, using B Coefficient a formula for prediction of ATP/ADP ratio could be obtained : ATP/ADP = -0.990 + 0.105 [NO].

## Discussion

**Figure 3:**
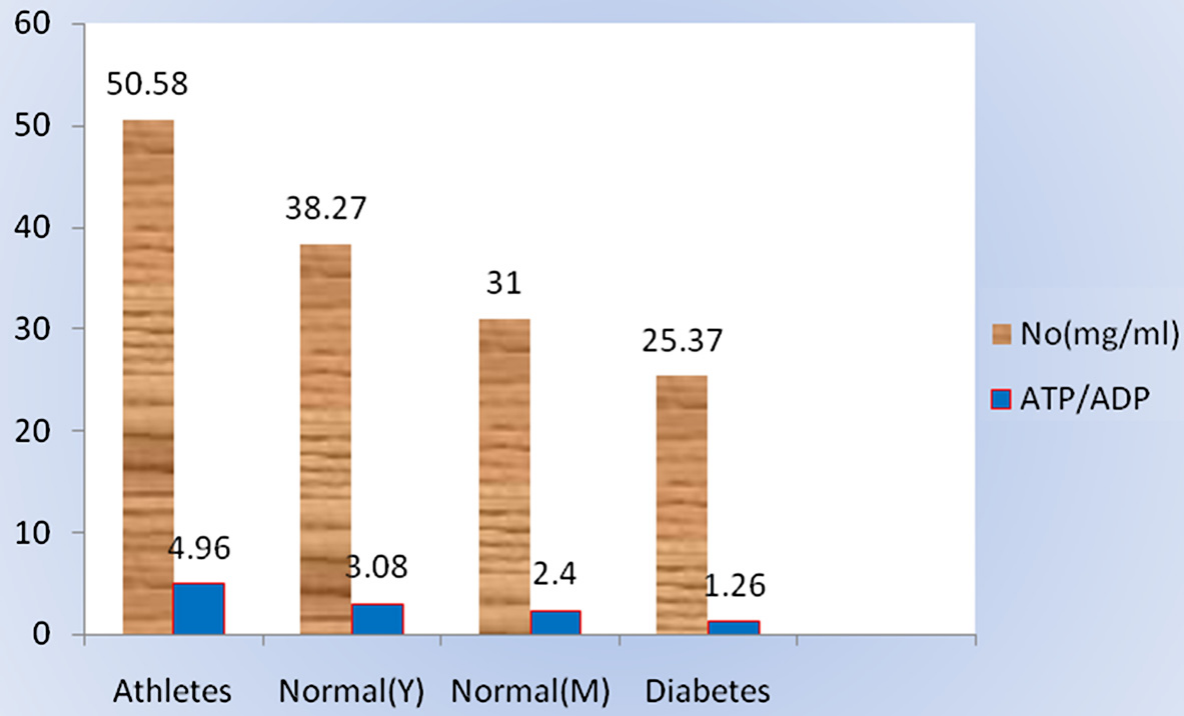
Correlation between NO and RBC ATP/ADP with bar curve.

**Figure 4:**
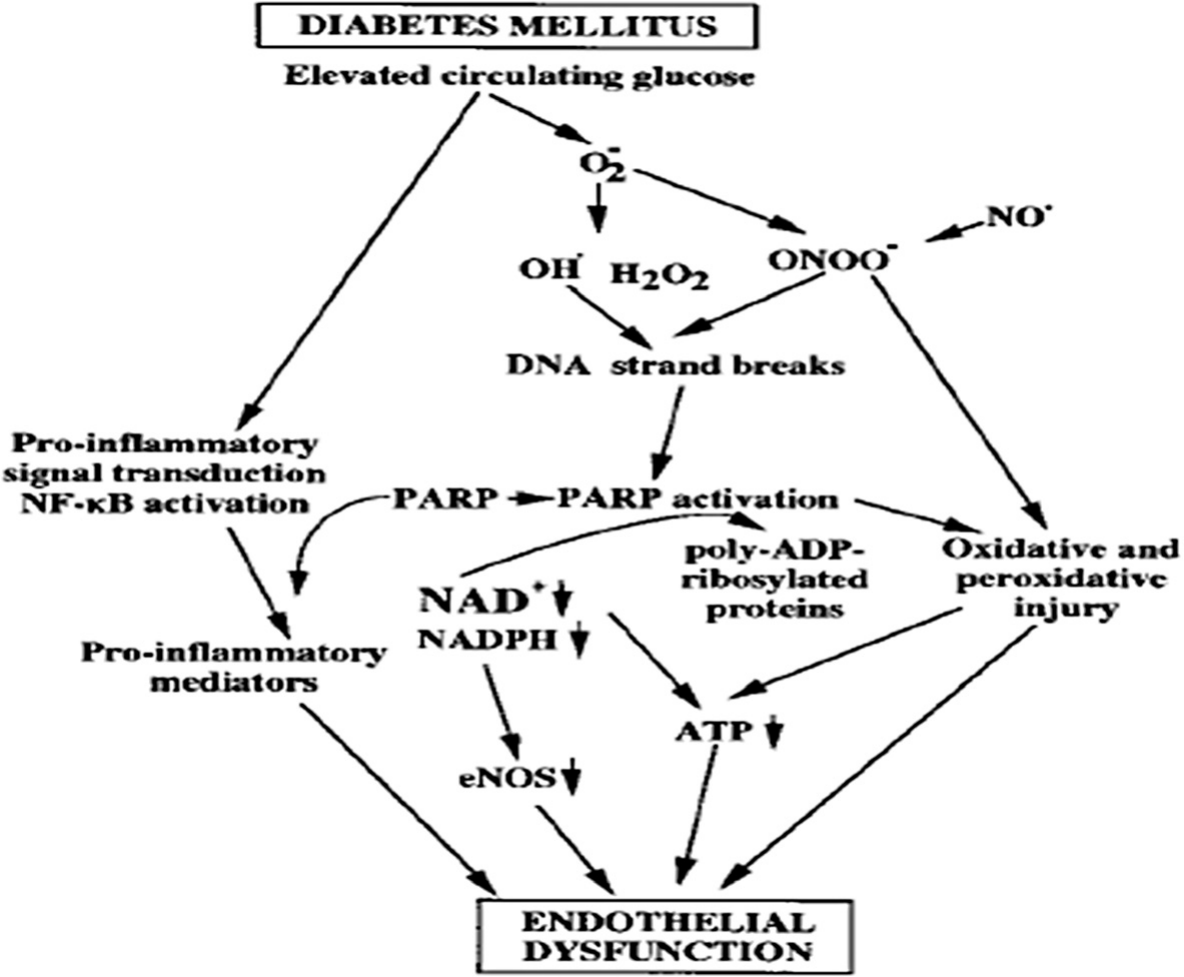
Proposed scheme for the potential mechanism of factors which contribute in endothelial dysfunctionin the diabetic myocardium leading to improvements in eNOS function.

**Figure 5:**
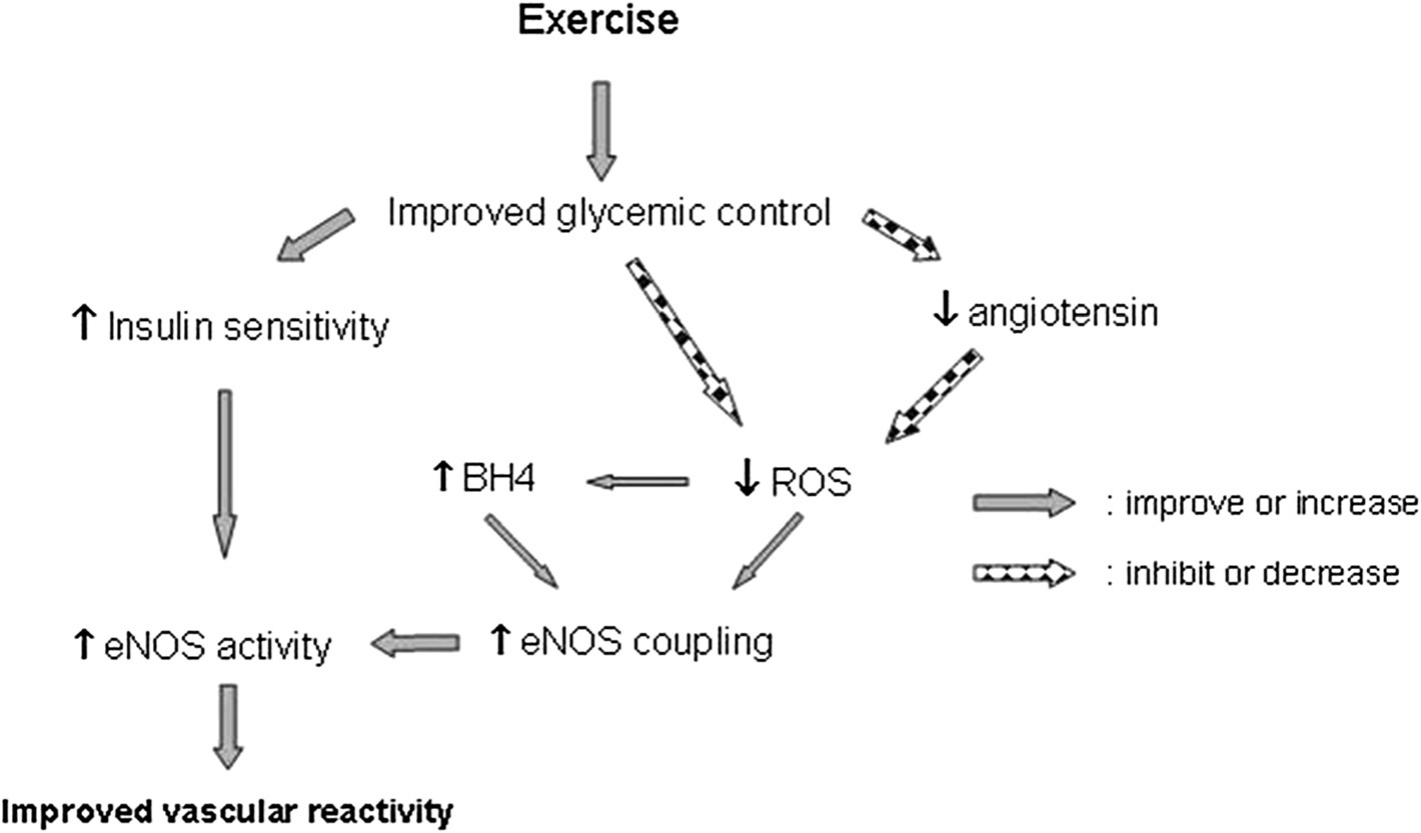
**Exercise induced alterations in the diabetic myocardium leading to improvements in eNOS function.** Decrease in level or inhibition of function, Increase in level or improvement of function. Improvements derived from increased insulin sensitivity are from the findings of Zhang et al. [46].

Nitric oxide (NO) is an omnipresent intercellular messenger in all vertebrates, modulating blood flow, thrombosis, and neural activity. Ellsworth et al. 1995; Sprague et al. 1996 had reported the erythrocyte, via its ability to release ATP, has been identified as a potential regulator of vascular resistance once released from the erythrocyte into the circulation, ATP can activate purinergic receptors, specifically those of the P2y subfamily, present on the vascular endothelium, resulting in the synthesis and release of NO. NO released abluminally interacts with vascular smooth muscle, resulting in its relaxation [36,37]. Controversially, Jaffrey J. etales have reported that inhibitory action of NO on ATP release from erythrocytes is the result of inactivation of one or more components of signal transduction pathway which contribute in ATP releasing in RBC. In this study we measured RBC ATP/ADP content and plasma NO between four groups (Normal (young) & (middle age), athletes, diabetes) (Figures 1, 2 Table 1). The present study investigate the relationship between of RBC ATP/ADP content and plasma NO level and the effect of exercise on RBC ATP/ADP content and plasma NO production. A positive significant correlation (Figure 3, Table 2) between plasma NO and ATP/ADP content of RBC among four groups has been observed. This observation suggests higher metabolic rate (ATP/ADP ratio) in athletes in comparison with normal and type2 diabetes. Other study reported that athletes have high ATP/ADP content in comparison with normal subject [38]. No correlation between RBC ATP/ADP content and plasma NO level has ever been reported. It is a sight of thought that plasma NO is a signal for inhibition of platelete aggregation and also is a signal for velocity in blood flowing which lead to wound healing. Previous works show that exposure of endothelial cells to oxidants leads to cellular energetic crisis, which is prevented by Poly (ADP ribose) polymerase (PARP) inhibition [39] this event simultaneously led to oxidative and peroxidative injury, decrease in NAD^+^, NADPH which make decrease in ATP production and eNOS activity which led to endothelial dysfunction (Figure 4). Sprague et al. (1996) reported that both rabbit and human erythrocytes release ATP in response to mechanical deformation produced by passage of erythrocytes through filters with an average pore size of 5 μm. Indeed, it has been reported that the RBCs from people with type 2 diabetes are less deformable than those RBCs obtained from healthy controls [40-42]. Have any role nitric oxide in cell metabolic rate? There is now clear evidence for a role of nitric oxide (NO) in mitochondrial biogenesis. NO donors increase the protein and mRNA expression of peroxisome proliferator-activated receptor coactivator 1 (PGC-1), nuclear respiratory factor 1 (NRF-1), and mitochondrial transcription factor A (mtTFA) in muscle cells and adipocytes of rodents [43], which are involved in coordinating mitochondrial biogenesis [44,45]. This process is dependent on the downstream messenger of NO, cGMP, via the activation of soluble guanylate cyclase (sGC) [43]. Furthermore, blocking the activation of sGC also blocks mitochondrial biogenesis induced by NO donors [43]. As mentioned above in athletes the ATP/ADP ratio and NO have a more positive significant in comparison with others, this due to exercise led to increase in ca^+2^ and then activation of nitric oxide synthase from a series of signal such as increase in ATP/ADP ratio, NADPH ,… and also decrease in reactive oxygen species(ROS) [46] (Figure 5).

**Table 2:** Pearson correlation between ATP/ADP and NO, this table showed that if NO 0.705 increased ATP/ADP level 1 unit increased

	**ATP/ADP& NO**	**Sig.**
**Pearson correlation**	0.705*	0.000
**Regression**	0.705	0.000

*. Correlation is significant at the 0.01 level (2-tailed).

Relationship between ATP/ADP and NO (Regression).

In conclusion, according to the result obtained in this investigation; the relationship between RBC ATP/ADP ratio and plasma NO is a positive significant and have a reciprocal correlation these factors was high in athletes and normal individuals in comparison with diabetic patients. It seems that ATP/ADP content may be considered as a main stimulator for NO secretion from endothelial cells, diabetes control and diabetic consequence such as stroke.

## Competing interest

The authors declare that they have no competing interests.

## Authors’ contributions

Nuredin Bakhtiary: had the main role in design, performance analysis of data, wrote of manuscript. Saman Hosseinkhani: supervisor of this project and help this idea both laboratory facilities and financial and deliver advantage guidance to progress this idea. Mohammad- reza mohajeri tehrani: the corresponding author of this project and had a benefit guidance in selecting individual( diabetes, controls and athletes) and approved this idea in shariati hospital gland & metabolism researcher center. Abbas Fallah: role in collecting athletic subjects form national Olympic commitee of Iran (judo national team). All authors read and approved the final manuscript.
